# Anti-Human Herpesvirus 6A/B IgG Correlates with Relapses and Progression in Multiple Sclerosis

**DOI:** 10.1371/journal.pone.0104836

**Published:** 2014-08-11

**Authors:** Isabel Ortega-Madueño, Marta Garcia-Montojo, Maria Inmaculada Dominguez-Mozo, Angel Garcia-Martinez, Ana Maria Arias-Leal, Ignacio Casanova, Rafael Arroyo, Roberto Alvarez-Lafuente

**Affiliations:** 1 Servicio de Análisis Clínicos. Hospital Clínico San Carlos, Instituto de Investigación Sanitaria del Hospital Clínico San Carlos (IdISSC), Madrid, Spain; 2 Servicio de Neurología. Hospital Clínico San Carlos, Instituto de Investigación Sanitaria del Hospital Clínico San Carlos (IdISSC), Madrid, Spain; National Institutes of Health, United States of America

## Abstract

**Objective:**

To analyze the titers of the IgG and IgM antibodies against human herpesvirus 6A/B (HHV-6A/B) in multiple sclerosis (MS) patients treated with different disease modified therapies (DMTs) along two-years of follow-up.

**Methods:**

We collected 2163 serum samples from 596 MS; for 301 MS patients a 2-years follow-up was performed. Serum samples of 337 healthy controls were also analyzed. Anti-HHV-6A/B IgG and IgM were analyzed by ELISA (Panbio).

**Results:**

We found that 129/187 (69.0%) MS patients with a decrease of the anti-HHV-6A/B IgG titers after 2-years with DMTs were free of relapses and progression vs. 46/113 (40.7%) of MS patients with an increase of the anti-HHV-6A/B IgG titers (p = 0.0000015); the higher significance was found for natalizumab. Furthermore, we found that anti-HHV-6A/B IgG titers reached their highest value two weeks before the relapse (p = 0.0142), while the anti-HHV-6A/B IgM titers reached their highest value one month before the relapse (p = 0.0344).

**Conclusion:**

The measurement of the anti-HHV-6A/B IgG titers could be a good biomarker of clinical response to the different DMTs. The increase of the anti-HHV-6A/B IgG and IgM titers predicts the upcoming clinical relapses. However, further longitudinal studies are needed to validate these results.

## Introduction

Multiple sclerosis (MS) is an inflammatory and degenerative neurological disease in which damage to the central nervous system causes widespread dysfunction [1]. Early in the course of MS, disease modifying therapies (DMTs), such as interferon-beta (IFN-beta), glatiramer acetate (GA) or natalizumab reduce the relapse rate and the rate of disability progression [2]–[4].

There are increasing evidences that a number of environmental factors are important in the development and course of MS. Although no virus or other environmental agents have been definitively implicated as a causative factor of MS, certain human herpesviruses (HHVs) have been linked with the development of MS [5], especially the Epstein-Barr virus (EBV) [6]–[8], and the formerly known as HHV-6 [9]–[11]; although some authors have described a possible relation between HHV-6B and MS [12], it appears that HHV-6A could be mainly associated with MS [13]–[15]. Different mechanisms have been proposed for these viruses in MS pathogenesis; but, for these viruses or for the other viruses or possible environmental factors that could be involved in MS, a relation with the evolution of the disease and the clinical response to the different DMTs should be demonstrated. Thus, the aim of this study was to analyze the titers of the IgG and IgM antibodies against HHV-6A/B in MS patients treated with different DMTs along two-years of follow-up.

## Materials and Methods

### Subjects

We collected 2163 serum samples from 596 MS patients in a prospective study (see Table 1). For 301 MS patients a 2-years longitudinal study was performed: a serum sample was collected prior the beginning of a DMT, and each three months (MS patients treated with natalizumab) or six months (MS patients treated with IFN-beta or GA) to complete, at least, two-years of follow up; a serum sample was also collected when the patient suffered a relapse (prior intravenous corticosteroids). Serum samples of 337 healthy controls were also included in the study. For MS patients we collected the following clinical data: the Expanded Disability Status Scale (EDSS) score prior the beginning of the DMT and two years later, and the number of relapses along the two-years of follow-up with the different DMTs.

**Table 1:** Clinical and demographic characteristics of the samples and subjects included in the study.

Serum samples of MS patients		2163
	In relapse (prior intravenous corticosteroids)	216
	In remission	1947
	Within the three months before and after a relapse	278
	% Serum samples collected without treatment	24.7
	% Serum samples collected during interferon beta treatment	27.7
	% Serum samples collected during glatiramer acetate treatment	24.3
	% Serum samples collected during natalizumab treatment	23.3
MS patients		596
	Females	384
	Males	212
	MS patients with at least two-years of follow-up*	301
	Relapsing-remitting MS patients	279
	Naïve patients	148
	Secondary progressive MS patients	22
	Age at the beginning of the study (years)	36.4
	Duration of the disease (years)	7.0
	Starting age of the disease (years)	29.4
	EDSS at the recruitment**	2.4
	MSSS at the recruitment**	4.0
	Number of relapses two years before starting the treatment	2.3
	MS patients treated with interferon beta	131
	MS patients treated with glatiramer acetate	89
	MS patients treated with natalizumab	81
	MS patients with one-year of follow-up	112
	MS patients with less than one-year of follow-up	127
	Discontinuations before completing 2-years of follow-up***	56
Healthy controls		337
Serum samples of healthy controls		337
Sex	Females	192
	Males	169
Age at the recruitment		37.6

*At least two-years of follow-up with the same disease modifying therapy (DMT) from a base line visit (serum sample extracted before starting the DMT) to a 24 months visit.

**EDSS: Expanded Disability Status Scale. MSSS: Multiple Sclerosis Severity Score.

***It was included: adverse events, pregnancy, drug withdrawal for lack of efficacy, and patient decision.

### Ethics Statement

This study was approved by our local Ethic Committee (“Comité Ético de Investigación Clínica del Hospital Clínico San Carlos”), and all the participants received and signed the written informed consent before the enrolment.

### Samples

One dry tube with blood was collected for each patient in each one of the visits. After collection of the whole blood, we allowed the blood to clot by leaving it undisturbed at room temperature along 30 minutes; then, we removed the clot by centrifuging at 1,500 × g for 10 minutes in a refrigerated centrifuge. Finally, the serum samples were aliquoted in 0.2 ml tubes, and the aliquots were frozen at −80°C during 32.6±14.1 before being analyzed (the mean time needed to complete one plate).

### Anti-HHV-6A/B IgG and IgM ELISA

Every serum sample was tested with two tests of Panbio (Inverness, Australia), for the detection of anti-HHV-6A/B IgG and IgM following the manufacturer instructions. In brief, serum antibodies of the IgG or IgM class, when present, combined with an HHV-6 antigen that was attached to the polystyrene surface of the microwells. Residual serum was removed by washing and peroxidase conjugated anti-human IgG or IgM was added. The microwells were washed and a colorless substrate system, tetramethylbenzidine/hydrogen peroxide (TMB/H2O2) was added. The substrate was hydrolyzed by the enzyme and the chromogen changed to a blue color. After stopping the reaction with acid, the TMB became yellow. The color intensity was directly related to the concentration of HHV-6 IgG or IgM antibodies in the test sample. Results were expressed in Panbio units (PU); they were calculated by multiplying the index value by 10 (the index value for each one of the samples = sample absorbance/cut-off value). Samples were analyzed in duplicate for each test, and doubtful samples, those that were between 9 and 11 Panbio units (PU) were tested again: a total of 60 serum samples (49 from MS patients and 11 from healthy controls) had titers between 9–11 PU; 3 of them (that belonged to 3 MS patients) were considered positive after the new analysis: one of them had a titer value of 10.8 PU and other two samples had 10.7 PU; after the new analysis, the samples had titers of 11.1 PU, 11.1 PU and 11.2 PU, respectively (the variation inter-assays was under 5%). The other doubtful serum samples were considered as negative.

### Detection of neutralizing antibodies (NAbs) against IFN-beta

NAbs were measured in the serum samples of those MS patients treated with IFN-beta through the cytopathic effect (CPE) assay at 6, 12, 18 and 24 months after starting the treatment, as it has been previously described [16]. The titers were calculated according to Kawade’s formula [17], and expressed in tenfold reduction unit (TRU). Samples were considered positives if titers were >20 TRU/ml.

### Statistical analysis

Odds ratios (O.R.) and exact 95 percent confidence intervals (C.I.) were calculated with standard microcomputer software: Epi Info v. 6.02 (CDC, Atlanta, USA) and SPSS Ver. 15.0 (SPSS Inc.). The chi-square or two-tailed Fisher’s exact test was used to test differences in categorical variables. Kruskall-Wallis analysis or the Wilcoxon rank-sum test was used to test differences in continuous variables. We considered statistically significant differences when p<0.05.

## Results

### Serology of HHV-6A/B in serum samples of MS patients and healthy controls

When we analyzed only those serum samples collected when MS patients were untreated (Table 2), we found that the anti-HHV-6A/B IgG prevalence was 98.0% (295/301) vs. 93.4% (315/337) in healthy controls (p = 0.005), and the mean titer was 26.8 PU vs. 23.1 PU in healthy controls (p = 0.00002). Regarding anti-HHV-6A/B IgM antibodies, the prevalence was 8.3% (25/301) vs. 5.6% (19/337) in healthy controls (p = 0.184) and the mean titer was 4.4 PU vs. 4.0 PU in healthy controls (p = 0.100).

**Table 2:** Prevalences and mean titers of the anti-HHV-6A/B IgG and IgM antibodies before and after 2-years of treatment with the different DMTs.

	Anti-HHV-6A/B IgG					
	Prevalences			Mean Titers (PU)		
DMTs	Before	2-years	p	Before	2-years	p
TOTAL	98.0% (295/301)	97.0% (292/301)	0.433	26.8	25.3	0.044
IFN-beta	96.9% (127/131)	96.9% (127/131)	1	24.3	24.1	0.439
GA	98.9% (88/89)	96.6% (86/89)	0.312	30.0	27.9	0.105
NTZ	97.5% (79/81)	97.5% (79/81)	1	27.4	24.3	0.033

	Anti-HHV-6A/B IgM					
	Prevalences			Mean Titers (PU)		
DMTs	Before	2-years	p	Before	2-years	p
TOTAL	8.3% (25/301)	5.3% (16/301)	0.145	4.4	3.6	0.003
IFN-beta	6.1% (8/131)	6.1% (8/131)	1	4.1	3.5	0.057
GA	11.2% (10/89)	6.7% (6/89)	0.295	4.8	4.2	0.136
NTZ	8.6% (7/81)	2.5% (2/81)	0.086	4.5	3.1	0.024

TOTAL: prevalence and mean titers of all the different disease modifying therapies (DMTs); IFN-beta: interferon beta; GA: glatiramer acetate; NTZ: natalizumab.

### Serology of HHV-6A/B and DMTs in MS patients

As can be seen in Table 2, we found a statistical significant reduction of the anti-HHV-6A/B IgG titers after 2-years with the different DMTs (p = 0.044), and above all, with natalizumab (0.033). When we analyzed the anti-HHV-6A/B IgM antibodies (Table 2), we found a trend for the reduction of the prevalence in MS patients treated with natalizumab, and a decrease of the mean titers with the different DMTs (p = 0.003); again, the greatest reduction was for natalizumab (p = 0.024).

### Serology of HHV-6A/B and clinical response to the different DMTs

**Figure 1:**
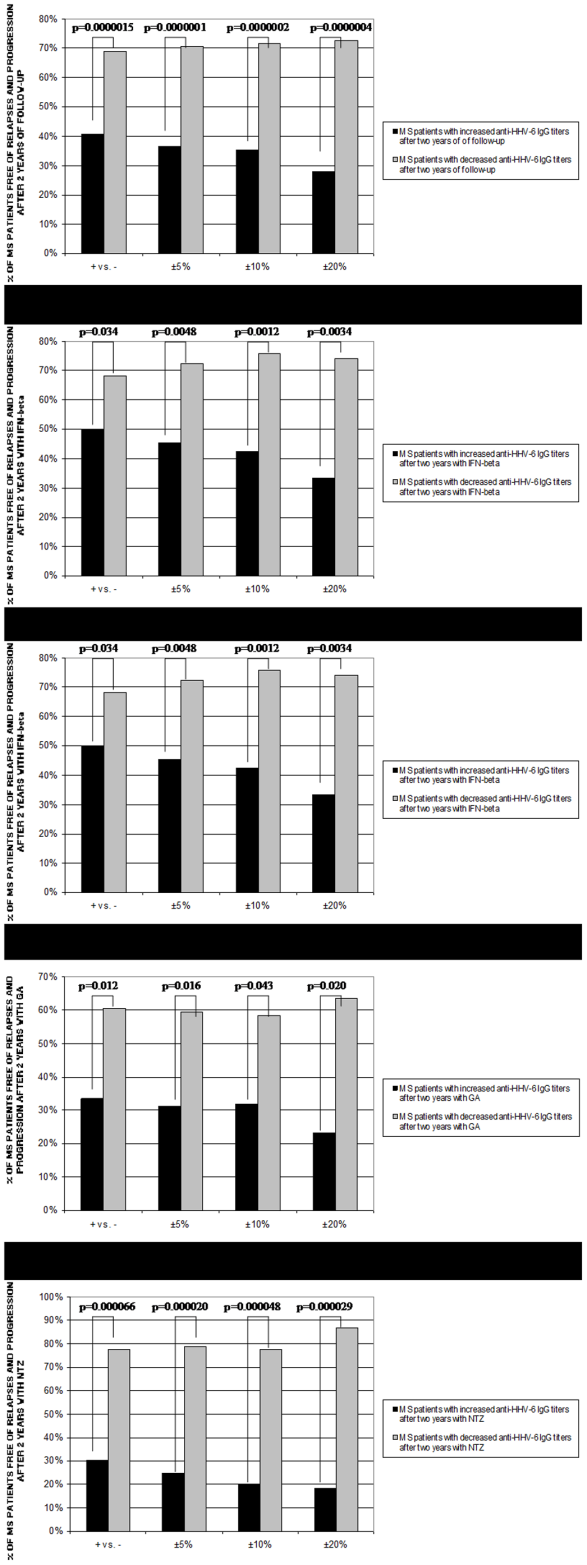
Percentage of MS patients free of relapses and progression after two years of follow-up from the 301 MS patients included in the longitudinal study. A. DMTs. B. interferon-beta (IFN-beta). C. IFN-beta (only in those MS patients that did not develop NAbs). D. glatiramer acetate (GA). E. natalizumab (NTZ). Four comparisons have been performed for each treatment: MS patients with increased anti-HHV-6A/B IgG titers vs. MS patients with decreased anti-HHV-6A/B IgG titers; MS patients with an increase of the anti-HHV-6A/B IgG titers >5% vs. MS patients with a decrease of the anti-HHV-6A/B IgG titers >5%; MS patients with an increase or decrease of the anti-HHV-6A/B IgG titers >10%; MS patients with an increase or decrease of the anti-HHV-6A/B IgG titers >20%.

As can be seen in Figures 1A–1E, we found a correlation between the variation of the anti-HHV-6A/B IgG titers and the clinical response (absence of relapses and progression after two-years of follow-up) in MS patients treated with the different DMTs: 129/187 (69.0%) MS patients with a decrease of the anti-HHV-6A/B IgG titers were free of relapses and progression vs. 46/113 (40.7%) of MS patients with an increase of the anti-HHV-6A/B IgG titers (p = 0.0000015); statistical significant differences were found for each one of the DMTs, although the higher significance was found for natalizumab: 45/58 (77.6%) vs. 7/23 (30.4%) (p = 0.00007; O.R. = 7.9), respectively. The percentage of clinical responders was greater among those MS patients with greater reductions of the anti-HHV-6A/B IgG titers, while the percentage of clinical responders in MS patients with an increase in their anti-HHV-6A/B IgG titers was lower when the percentage of increase was greater (Figures 1A–1E).

The mean variation of the anti-HHV-6A/B IgG titers (around 20% of increase or decrease) was lower than the mean variation of the anti-HHV-6A/B IgM titers (more than 30% of increase or decrease); no statistical significant correlations were found between the clinical response and the variation of the anti-HHV-6A/B IgM titers after two years with the different DMTs, only a trend for natalizumab when we compared MS patients with increases or decreases >30% (37.5% vs. 69.2%, respectively, p = 0.089). Finally, when we compare those MS patients with an increase of the anti-HHV-6A/B IgG titers>20% and with an increase of the anti-HHV-6A/B IgM titers>30% (both above the mean variation of increase) with those MS patients with a decrease of the anti-HHV-6A/B IgG titers>20% and with a decrease of the anti-HHV-6A/B IgM titers>30% (both above the mean variation of decrease), for all the DMTs, we found a high statistical significance (p = 0.00002; O.R. = 39.9): 1/10 MS patients was free of relapses and progression vs. 31/38 MS patients, respectively.

An association was also found between the increase of the anti-HHV-6A/B IgG titers and the clinical non-response (MS patients with at least two relapses and/or an increase of at least one point in the EDSS score after two years of follow-up with the different DMTs): 35/113 (31.0%) MS patients with an increase of the anti-HHV-6A/B IgG titers were non-responders vs. 33/187 (17.6%) MS patients with a decrease of the anti-HHV-6A/B IgG titers (p = 0.0076; O.R. = 2.1); furthermore, 22/54 (40.7%) MS patients with an increase >20% of the anti-HHV-6A/B IgG titers were non-responders vs. 11/77 (14.3%) MS patients with a decrease >20% (p = 0.0006; O.R. = 4.1). Again, the greater statistical significance was for natalizumab (p = 0.018; O.R. = 3.9).

### Serology of HHV-6A/B in MS patients treated with IFN-beta without NAbs

The 32.8% (43/131) of MS patients treated with IFN-beta were positive for NAbs at least once along the two years of follow-up. When we only considered those MS patients treated with IFN-beta that did not develop NAbs in the 2-years of follow-up (Figure 1.C), we found that 16/38 (42.1%) MS patients with an increase of the anti-HHV-6A/B IgG titers were clinical responders vs. 39/50 (78%) MS patients with a decrease of the anti-HHV-6A/B IgG titers (p = 0.0006; O.R. = 4.9); furthermore, 4/21 (19%) MS patients with an increase of the anti-HHV-6A/B IgG titers >20% were clinical responders vs. 13/16 (81.2%) MS patients with a decrease of the anti-HHV-6A/B IgG titers >20% (p = 0.0002; O.R. = 18.4).

### Serology of HHV-6A/B and EDSS

**Figure 2:**
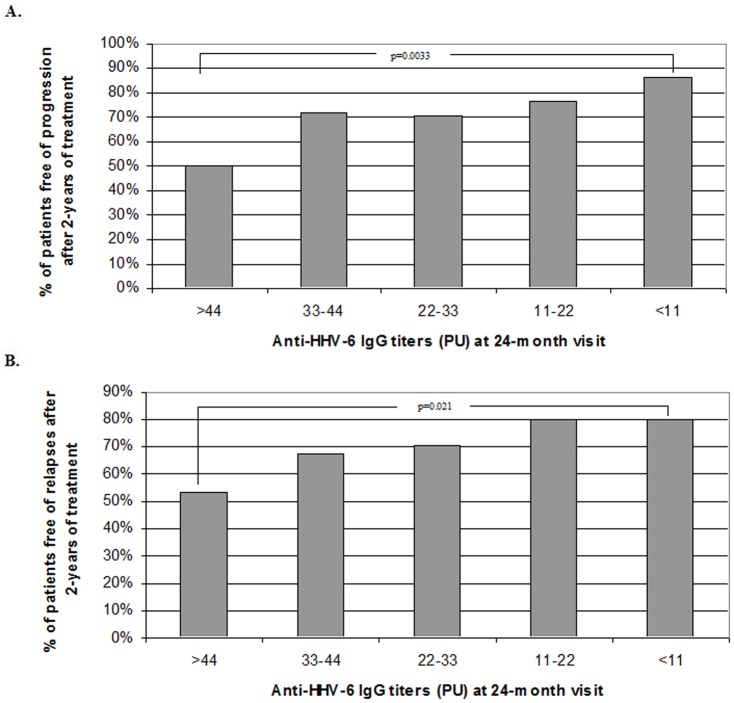
Association between the anti-HHV-6A/B IgG titers at the 2-years visit with the progression of the disease (A) and the activity of the disease (B) after two years of treatment with the different DMTs. A. 9/18 MS patients with titers >44 PU after 2-years of treatment were free of progression vs. 39/54 with titers between 33–44 PU vs. 74/104 with titers between 22–33 PU vs. 82/108 with titers between 11–22 PU vs. 15/17 with titers <11 PU. B. 10/18 MS patients with titers >44 PU after 2-years of treatment were free of relapses vs. 37/54 with titers between 33–44 PU vs. 74/104 with titers between 22–33 PU vs. 80/108 with titers between 11–22 PU vs. 14/17 with titers <11 PU.

Only the 28.2% (53/188) of MS patients that did not experienced an increase in the EDSS score (the EDSS was equal or lower after 2-years of treatment) had an increase of the anti-HHV-6A/B IgG titers; however, among those MS patients that experienced an increase in the EDSS after two years of treatment, the 62.9% (44/70) had an increase of the anti-HHV-6A/B IgG titers (p = 0.0000003; O.R. = 4.3). Furthermore, as we can see in Figure 2.A, the lower anti-HHV-6A/B IgG titers at 24-months visit, the greater the percentage of patients free of progression after two years of treatment.

### Serology of HHV-6A/B and relapses

**Figure 3:**
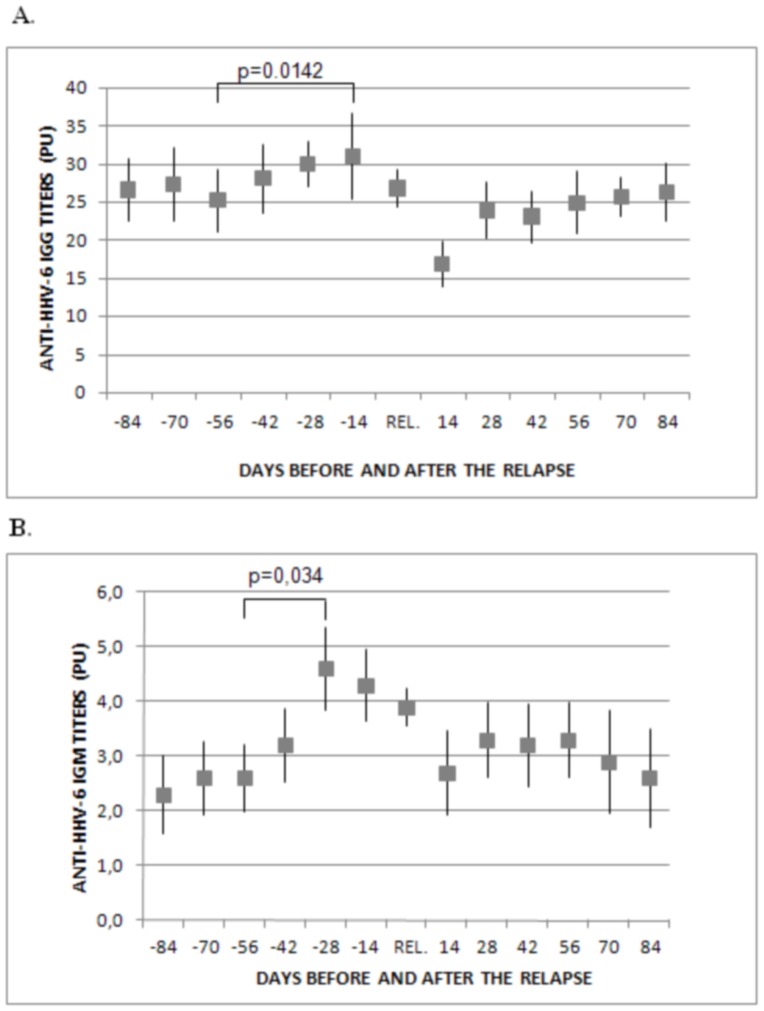
Anti-HHV-6A/B IgG and IgM titers in 216 serum samples collected in relapse (REL.) and in 278 serum samples collected three months before and after the relapse (n = 21 at day–84, n = 20 at day –70, n = 23 at day –56, n = 23 at day –42, n = 25 at day –28, n = 31 at day –14, n = 26 at day 14, n = 21 at day 28, n = 22 at day 42, n = 24 at day 56, n = 23 at day 70, n = 19 at day 84). A. Anti-HHV-6A/B IgG response. B. Anti-HHV-6A/B IgM response.

For the study of the anti-HHV-6A/B IgG and IgM response in relapses we included 216 serum samples collected in relapse and 278 serum samples collected within the three months before and after a relapse, from the total of 2163 serum samples collected from the 596 MS patients included in the study (Table 1). As can be seen in Figure 3.A, when we analyzed the anti-HHV-6A/B IgG response in relapses, we found that two months before the relapse the IgG titers began to increase and they reached their highest value two weeks before the relapse (p = 0.0142); then, they began to decrease and reached the lowest value two weeks after the relapse. Similarly, the anti-HHV-6A/B IgM titers increased around two months before the relapse, they reached their highest value one month before the relapse (p = 0.0344), two weeks before the IgG response (Figure 3.B). Furthermore, similarly to what happened to the progression, we found that higher titers of anti-HHV-6A/B IgG at 24-month visit was associated with a higher likelihood of relapses during the two years of treatment (Figure 2.B).

## Discussion

The results of this study show a good correlation between the variation of the anti-HHV-6A/B IgG titers and the clinical response to the different DMTs in MS patients after two years of follow up (Figures 1A–1E). We found that MS patients with higher titers after two-years of treatment had higher likelihood of progression and relapses (Figure 2); and furthermore, we have found an association between the increase of the anti-HHV-6A/B IgG and IgM titers (Figure 3) with the upcoming relapses (however, as IgM antibodies to HHV-6 from Panbio can cross react with a number of other viral antigens such as CMV and EBV, the serology of these viruses should be also analyzed).

In previous studies of our group, we have reported that the presence of HHV-6A/B DNA in blood and serum during IFN-beta treatment could be a good marker of poor response [18]–[20]. Similar results were found by other authors in MS patients treated with IFN-beta when serum cell-free DNA of HHV-6A/B was analyzed [21]. IFN-beta is a cytokine naturally expressed in response to viral infections [22], however, its mechanism of action in MS is not completely understood, but it has been speculated that, besides its immunomodulatory properties, the efficacy of IFN-beta may be related to its antiviral properties [23]. However, it has been observed consistently that a proportion of patients (2% to 47%) develop NAbs directed against IFN beta as a consequence of the treatment [24], [25]. In our study, the 32.8% of MS patients was positive for NAbs; as NAbs are associated with a loss of efficacy of IFN-beta treatment and a reduced bioavailability, they appeared at any time of the four scheduled visits and while some NAbs positive patients remained positive along the IFN-beta treatment, others patients became negative, we decided to evaluate the variation of the anti-HHV-6A/B IgG titers only in an homogenous cohort of MS patients without NAbs. As we have previously mentioned, the correlation between the anti-HHV-6A/B IgG variation and the clinical response was greatly enhanced when we only considered those MS patients treated with IFN-beta that did not develop NAbs (p = 0.0006) instead of the whole population of IFN-beta treated MS patients (p = 0.034). Therefore, it would be important to distinguish between MS patients with and without NAbs in future studies of other viruses and possible biomarkers in MS patients treated with IFN-beta, to obtain more accurate and reliable results.

There are not previous studies on GA and viruses in MS. GA is an acetate salt of a random polymer of four amino acids that shares some cross-reactivity with myelin basic protein (MBP) [26]. The mechanisms by which GA exerts its effects in MS patients are not fully elucidated: generating suppressor cells, inducing tolerance, expanding regulatory T-cell populations, and altering antigen-presenting cells have all gained favor for a time as the mechanism for the immunomodulatory effects of GA [27]. In our study, we found a significant correlation between the reduction of the anti-HHV-6A/B IgG titers after 2-years of treatment and the clinical response.

Natalizumab is a humanized monoclonal antibody that selectively binds to the α4-integrin component of adhesion molecules found on lymphocytes, monocytes, and eosinophils; thus, natalizumab inhibits the interaction of α4β1 with VCAM-1, and because VCAM-1 is expressed on inflamed cerebrovascular endothelial cells, α4β1 is believed to be the critical target of natalizumab in preventing leukocyte migration into the central nervous system in MS [28]. Furthermore, it has been published that the treatment leads to a significant decrease in serum IgM and IgG levels in patients with MS; these findings might support the hypothesis that natalizumab interferes with homing of B cells, possibly leading to impaired differentiation into plasma cells and subsequently disturbed immunoglobulin synthesis [29]. In our study, we have also found a significant decrease of the anti-HHV-6A/B IgG and IgM titers after two-years of treatment; but, what it is most interesting, we have found a high correlation between the variation of the anti-HHV-6A/B IgG titers and the clinical response to this treatment (Figure 1.E).

The relationship with the clinical response for all the DMTs was found both for the progression and for the relapses. Furthermore, the increase of the anti-HHV-6A/B IgG and IgM titers predicts the upcoming clinical relapses. Recently, Simpson et al. [30] published that HHV-6A/B infection or the immune response to HHV-6A/B antigens may have an effect on the risk of MS relapses, since the observed effect was directly related to anti-HHV-6A/B IgG titers and may indicate that either HHV-6A/B infection or factors associated with an altered humoral immune response to HHV-6A/B may have an effect on MS clinical course.

Then, we can conclude that the measurement of the anti-HHV-6A/B IgG titers could be a good biomarker of clinical response to the different DMTs. However, without magnetic resonance imaging (MRI) data, we really don’t have a full picture about disease activity and therapeutic responses of the MS patients included in this study; further longitudinal studies that include MRI data are needed to validate these results.
